# Biocatalytic Production of Trehalose from Maltose by Using Whole Cells of Permeabilized Recombinant *Escherichia coli*

**DOI:** 10.1371/journal.pone.0140477

**Published:** 2015-10-13

**Authors:** Zhaojuan Zheng, Ying Xu, Ye Sun, Wending Mei, Jia Ouyang

**Affiliations:** 1 College of Chemical Engineering, Nanjing Forestry University, Nanjing 210037, People’s Republic of China; 2 Jiangsu Key Lab of Biomass-based Green Fuels and Chemicals, Nanjing 210037, People’s Republic of China; 3 Key Laboratory of Forest Genetics and Biotechnology of the Ministry of Education, Nanjing 210037, People’s Republic of China; Imperial College London, UNITED KINGDOM

## Abstract

Trehalose is a non-reducing disaccharide, which can protect proteins, lipid membranes, and cells from desiccation, refrigeration, dehydration, and other harsh environments. Trehalose can be produced by different pathways and trehalose synthase pathway is a convenient, practical, and low-cost pathway for the industrial production of trehalose. In this study, 3 candidate *treS* genes were screened from genomic databases of *Pseudomonas* and expressed in *Escherichia coli*. One of them from *P*. *stutzeri* A1501 exhibited the best transformation ability from maltose into trehalose and the least byproduct. Thus, whole cells of this recombinant *E*. *coli* were used as biocatalyst for trehalose production. In order to improve the conversion rate of maltose to trehalose, optimization of the permeabilization and biotransformation were carried out. Under optimal conditions, 92.2 g/l trehalose was produced with a high productivity of 23.1 g/(l h). No increase of glucose was detected during the whole course. The biocatalytic process developed in this study might serve as a candidate for the large scale production of trehalose.

## Introduction

Trehalose (α-D-glucopyranosyl α-D-glucopyranoside) is a non-reducing disaccharide formed by an α-1,1 linkage of two glucose molecules. In nature, trehalose is synthesized within insects, yeasts, and plants, especially those living in extreme environment [1, 2]. Because trehalose can protect proteins, lipid membranes, and cells from desiccation, refrigeration, dehydration, and other harsh environments, this disaccharide plays an important role in pharmaceuticals, foods, and cosmetics filed [3–5].

**Fig 1:**
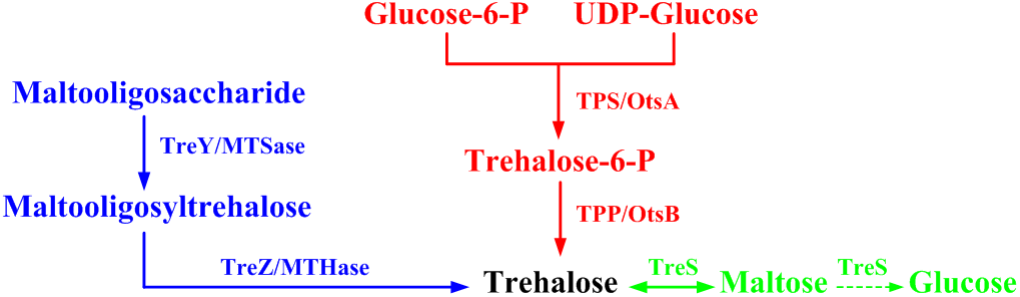
Biosynthesis of trehalose by three different pathways.

Because of the widely utilization, various methods, both chemical and biotechnological routes, have been developed for trehalose production [5]. As early as in 1954, the chemical synthesis of trehalose was created, but the method was difficult to realize industrialization. Also, the chemical transformation had some disadvantages, such as low production rate, coproduction of other trehalose anomers, and difficult downstream separation [6, 7]. In the past decades, several biological methods for the production of trehalose have been reported. The biosynthesis of trehalose is mainly consisted of three different pathways (Fig 1) [5, 8, 9]. The most widely distributed pathway is the TPS/TPP or OtsA/OtsB pathway. It involves two enzymes, trehalose-6-phosphate synthase (TPS or OtsA) and trehalose-6-phosphate phosphatase (TPP or OtsB). The TPS transfers glucose unit from UDP or GDP-glucose to glucose-6-phosphate, forming the intermediate trehalose-6-phosphate, which is then hydrolyzed into trehalose by TPP [10–12]. Another pathway for trehalose synthesis includes two other enzymes, maltooligosyl trehalose synthase (MTSase or TreY) and maltooligosyl trehalose trehalohydrolase (MTHase or TreZ). The first enzyme acts on maltooligosaccharide or starch, and turns the α-1,4 linkage of the terminal disaccharide at reducing end into α-1,1 linkage. Thus the second enzyme releases the trehalose residue from the lower molecular weight maltooligosaccharide [13, 14]. Finally, a directly pathway is strictly dependent on trehalose synthase (TreS). Although TPS/TPP and TreY/TreZ pathways are more demonstrated for trehalose production, more attentions have been turned to the TreS pathway in recent years. In this pathway, trehalose is produced from inexpensive maltose in one step with or without the by-product, glucose. This method represents a convenient, practical, and low-cost pathway for the industrial production of trehalose [15–18].

Since TreS was first demonstrated in *Pimelobacter* sp. R48, an increasing number of *treS* genes from various strains, including *Thermomonospora curvata*, *Thermus aquaticus*, *Picrophilus torridus*, *Pseudomonas stutzeri*, *Pseudomonas putida*, *Arthrobacter auresce*, *Enterobacter hormaechei*, *Meiothermus ruber*, and other strains, had been identified, heterologously expressed, purified and characterized [16–23]. By comparing the reported TreSs, it revealed that their molecular masses, activities toward trehalose, and other enzymatic properties and biochemical characteristics, were diverse. Although many TreSs have been reported, compared with TPS/TPP and TreY/TreZ pathways, trehalose production using the TreS pathway is rare and the practical scale is not large enough. Discovery of new TreS and optimization of conversion process to satisfy the scale-up production of trehalose from maltose is still urgent.

Genome mining is an effective approach for searching new enzymes from genomic databases, and many successful applications of this strategy had been applied [24–26]. *Pseudomonas* is an opportunistic pathogen, which can use multiple strategies to cope with severe environments, such as water-limiting conditions. One such strategy is biosynthesis of trehalose [8, 27]. Nowadays, dozens of different *Pseudomonas* species had been sequences and annotated. Thereby, in the current study, candidate TreSs were screened from genomic databases of *Pseudomonas* by genome mining instead of traditionally identifying enzymes from organisms. Finally, one TreS from *P*. *stutzeri* A1501 with high ability toward maltose was identified and expressed in *Escherichia coli*. And a process utilizing permeabilized whole cells of engineered *E*. *coli* as the biocatalyst was developed for efficient production of trehalose from maltose.

## Materials and Methods

### Chemicals

Maltose, glucose, trehalose, isopropyl-β-D-thiogalactopyranoside (IPTG), and ampicillin were purchased from Sigma. Restriction enzymes, T4 DNA ligase, and standard proteins used as SDS-PAGE marker were procured from Fermentas (Lithuania). FastPfu DNA polymerase was obtained from Transgen Biotech (China). All other chemicals were of analytical grade and commercially available.

### Cloning and expression of *treS*

Three *treS* genes were selected and synthesized by Generay Biotech Co. (Shanghai, China) with addition of two restriction sites, *Hind* III and *EcoR* I, to the forward and reverse end, respectively. The synthetic product was firstly ligated to pEasy-Blunt vector (Transgen Biotech, China), and the resulting recombinant plasmid was designated as pEasy-Blunt-*treS*. Both pEasy-Blunt-*treS* and pETDuet-1 (Novagen) were digested with *Hind* III and *EcoR* I, and the *treS* gene was cloned into the multiple cloning sites of pETDuet-1 to generate the recombinant expression plasmid pETDuet-*treS*. After confirmed by DNA sequencing, pETDuet-*treS* was transformed into *E*. *coli* BL21(DE3) for expression.

### Recombinant protein production and purification

The *E*. *coli* BL21(DE3) harboring pETDuet-*treS* was designated as *E*. *coli* BL21(pETDuet-*treS*). The strain was inoculated into LB medium supplemented with 100 μg/ml ampicillin and grown at 37°C in a shaker at 200 rpm. When the culture reached an optical density of 0.6~0.8 at 600 nm, IPTG was added to a final concentration of 1.0 mM, and the incubation was continued for another 8 h at 16°C. The cells were harvested by centrifugation and resuspended in phosphate buffer solution (PBS, 50 mM, pH 7.4) followed by sonication and centrifugation at 10,000 × g for 15 min at 4°C to remove insoluble cell debris. The supernatant was used as crude cell extract. The crude enzyme was filtered and loaded onto a Ni-NTA affinity chromatography column (GE healthcare, HisTrap^TM^ HP). The purification was performed with gradient elution by using binding buffer (20 mM sodium phosphate, 500 mM sodium chloride, and 20 mM imidazole [pH 7.4]) and elution buffer (20 mM sodium phosphate, 500 mM sodium chloride, and 500 mM imidazole [pH 7.4]). Both the crude and purified enzymes were analyzed by SDS-PAGE. Protein concentrations were determined by the method of Bradford using bovine serum albumin as a standard.

### Preparation of permeabilized cells

After induction, the *E*. *coli* BL21(pETDuet-*treS*) cells were suspended in PBS and the permeabilizing reagent was added. After permeabilization, the cells were collected by centrifugation (10,000 × *g*, 15 min) at 4°C and resuspended in PBS. The permeabilized cell suspension was used as biocatalyst for trehalose synthesis in the following biotransformation reactions.

### Biotransformation by whole cells of permeabilized recombinant *E*. *coli*

To optimize the biotransformation conditions, the reactions were performed in 50 ml flasks with 10 ml of the reaction mixtures containing CKBB buffer (0.6008 g citric acid, 0.3893 g KH_2_PO_4_, 0.1769 g boric acid, and 0.5266 g barbitone dissolved in 1000 ml distilled water, pH 6.5), permeabilized recombinant cells, and maltose under different conditions. The optimum pH was assayed by incubating the cells with 50 g/l maltose in CKBB buffer at pH 4.0~12.0, respectively. The optimum temperature was determined at 15~55°C and cell concentrations were 9~45 g dry cell weight (DCW)/l. After reactions, the samples were heated to 100°C and centrifuged. The concentration of sugars in the resulting supernatants was quantitatively analyzed by high performance anion exchange chromatography and pulsed ampere detector (HPACE-PAD).

### Activity assay of recombinant TreS

The activity of TreS was assayed by measuring the amount of trehalose produced from maltose. The reaction was performed in a mixture containing the TreS solution and 5% maltose in CKBB buffer (pH 8.0) at 35°C for 1 h. The reaction was terminated by heating at 100°C for 10 min and centrifuged. Products released by TreS were quantified by IC using a Dionex3000 system. One unit (U) of TreS was defined as the amount of enzyme required to produce 1 μmol trehalose per min under the specified conditions.

### Analytical procedures

Trehalose, glucose and maltose were assayed by HPACE-PAD. The analysis was performed on a CarboPac^TM^10 column with a gradient elution of 200 mM NaOH and 500 mM NaAc as the mobile phase: 0 ~ 7 min, 75% water and 25% 200 mM NaOH; 7 ~ 15 min, 90% 200 mM NaOH and 10% 500 mM NaAc solution; 15 ~ 30 min, 75% water and 25% 200 mM NaOH. The column was set at 30°C, and the flow rate was 0.3 ml/min.

## Results

### Screening, expression and purification of TreS

**Fig 2:**
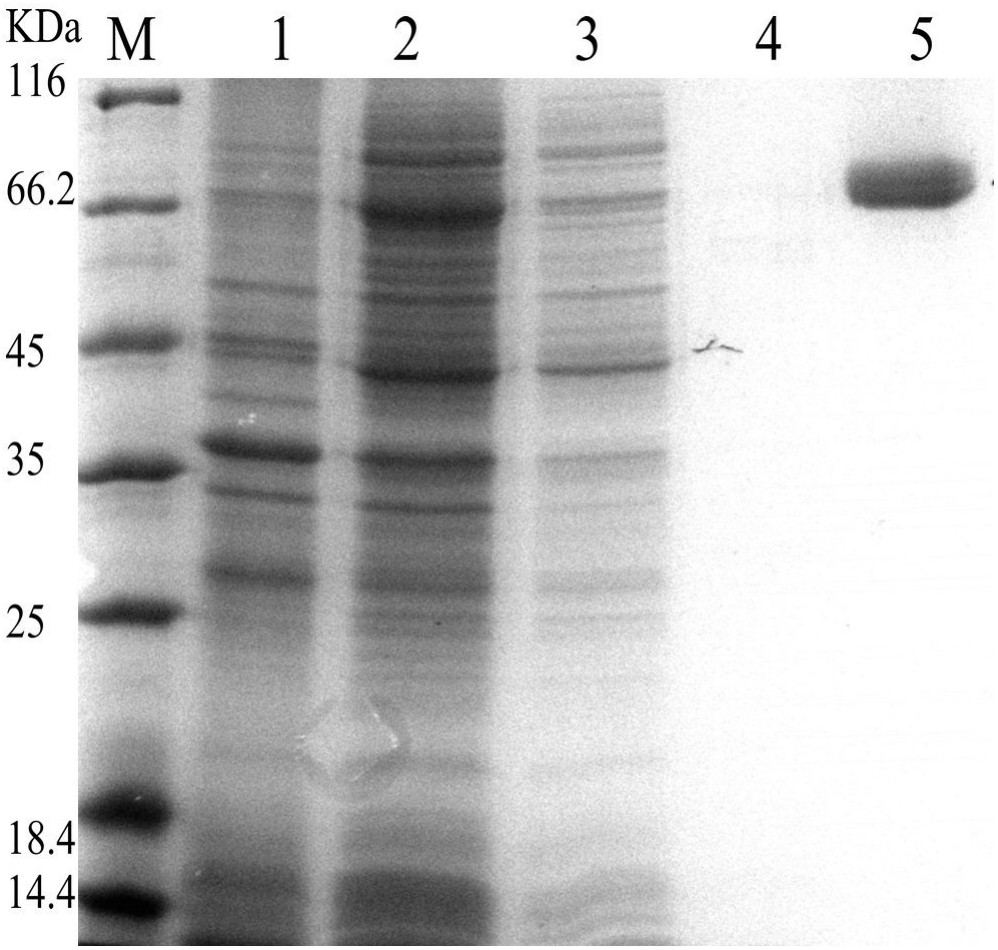
SDS-PAGE analysis of the recombinant TreS. Lane M, protein molecular weight markers; lane 1, the crude extract of the recombinant *E*. *coli* BL21 harboring pETDuet-1; lane 2, the crude extract of the recombinant *E*. *coli* BL21 harboring pETDuet-*treS*; lane 3, the sample eluted with 80% binding buffer and 20% elution buffer; lane 4, the sample eluted with 60% binding buffer and 40% elution buffer; lane 5, purified TreS eluted with 40% binding buffer and 60% elution buffer.

Genomic analysis of *Pseudomonas* showed numerous putative *treS* genes present on their genomes. Most of *treS* genes shared highly conserved amino acid sequence. After screening by sequence alignment, three putative *treS* genes (GeneID 5095109 and 5094839 from *P*. *stutzeri* A1501; GeneID 1042130 from *Pseudomonas putida* KT2440) were selected. These three genes were significantly different in length and similarity, and were thus chosen as potential biocatalyst for further studies. All of them were synthesized and expressed in *E*. *coli* using pETDuet-1 in order to identify their detailed characterics. Compared with the other two recombinant strains, *E*. *coli* harboring the 5095109 exhibited the best transformation ability from maltose into trehalose and the least byproduct (data not shown). Therefore, this recombinant protein was purified by Ni^2+^ affinity chromatography and detected by SDS-PAGE (Fig 2). The specific activity of purified recombinant TreS was 32.8 U/mg, indicating a 14.3-fold increase in specific activity from the crude extract.

### Effects of permeabilization treatment on trehalose production

In comparison with free enzymes, whole-cell biocatalysts are much more convenient for utilization and less expensive for preparation. However, the cell membrane often retards the substrate entry and prevents the product release. To improve trehalose production, permeabilization of induced *E*. *coli* BL21(pETDuet-*treS*) were carried out via chemical treatment. Different permeabilizing reagents, including chloroform, ethanol, EDTA, and toluene, were used to treat cells. The effects of permeabilizing reagents on trehalose production were shown in Table 1. Among them, chloroform and toluene resulted in much higher conversion yield, about 100-fold, than the control. In contrast, EDTA and ethanol had negative effects on trehalose production. Considering the conversion efficiency, chloroform was chosen to treat recombinant cells for further study.

**Table 1:** Effects of permeabilization treatment on the conversion rate of maltose to trehalose by various reagents.

Number	Permeabilizing reagents	Dosage (w/v)	Conversion rate of maltose to trehalose (%)
**1**	None	0%	0.4
**2**	Chloroform	2.0%	55.4
**3**	Ethanol	10.0%	0.0
**4**	EDTA	0.2%	0.0
**5**	Toluene	2.0%	39.8

The concentration of permeabilizing agent and the treatment time had a great influence on trehalose production. As shown in Table 2, when the concentration of chloroform increased from 0.1% to 1%, the conversion yield of maltose to trehalose was improved obviously. However, when the concentration of chloroform reached 4%, a visible decrease in the yield of trehalose was observed. This might be attributed to TreS inactivation because of a high concentration of chloroform. Effects of treatment time were also investigated. Under low concentration of chloroform, extension of time from 10 min to 60 min had positive effect. On the contrary, when concentration of chloroform was above 1%, longer time resulted in lower conversion yield. Therefore, 1% of chloroform dosage and 20 min for permeabilization were chosen in the following experiments.

**Table 2:** Effects of chloroform dosageon and treatment time on the conversion rate of maltose to trehalose.

Treatment time (min)	Conversion rate of maltose to trehalose (%)				
	0.1% chloroform	0.5% chloroform	1.0% chloroform	2.0% chloroform	4.0% chloroform
**0**	0.63±0.03	0.61±0.02	0.60±0.04	0.63±0.03	0.58±0.04
**10**	0.65±0.15	41.63±1.19	45.20±1.11	53.02±1.29	48.82±0.87
**20**	4.23±0.15	44.58±1.06	**55.85**±**2.50**	47.99±1.51	35.86±1.24
**30**	8.63±0.09	47.25±1.17	53.04±0.58	45.41±1.29	26.89±0.96
**60**	23.03±2.63	51.41±0.64	49.59±0.27	35.30±1.17	22.06±1.69

### Optimization of the biocatalysis conditions

**Fig 3:**
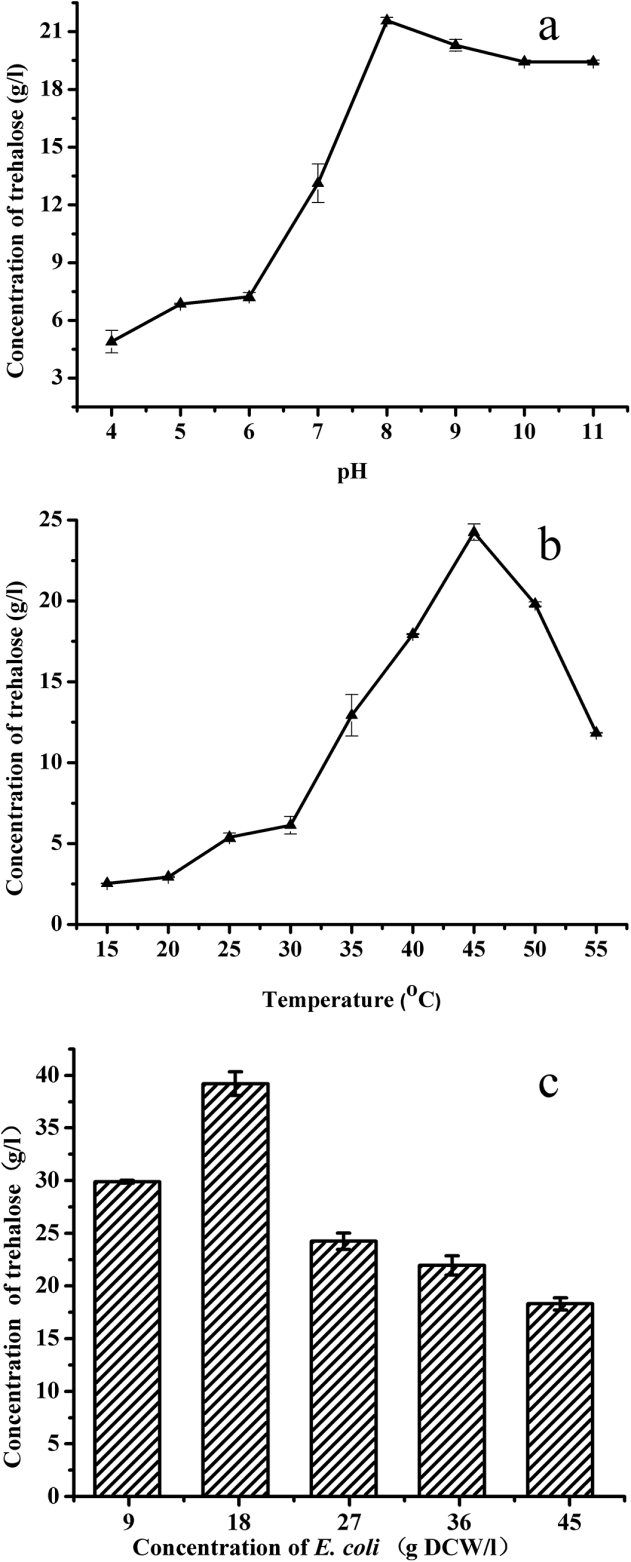
Optimization of biocatalysis conditions by using permeabilized cells. (a) pH; (b) Temperature; (c) Concentration of permeabilized cells.

To achieve higher conversion efficiency, the whole-cell biocatalytic reaction was optimized. The influence of reaction pH on trehalose production was determined in the range of pH 4 to 12. As shown in Fig 3A, after biocatalysis for 1 h, the highest production was detected at pH 8.0. To investigate the effect of reaction temperature on trehalose production, the reaction was carried out at different temperatures and pH 8.0. As shown in Fig 3B, the highest production was obtained at 45°C. The effect of the biocatalyst concentration was also investigated with 9~45 g DCW/l. As shown in Fig 3C, the highest conversions rate of maltose was observed at 17.7 g DCW/l.

**Fig 4:**
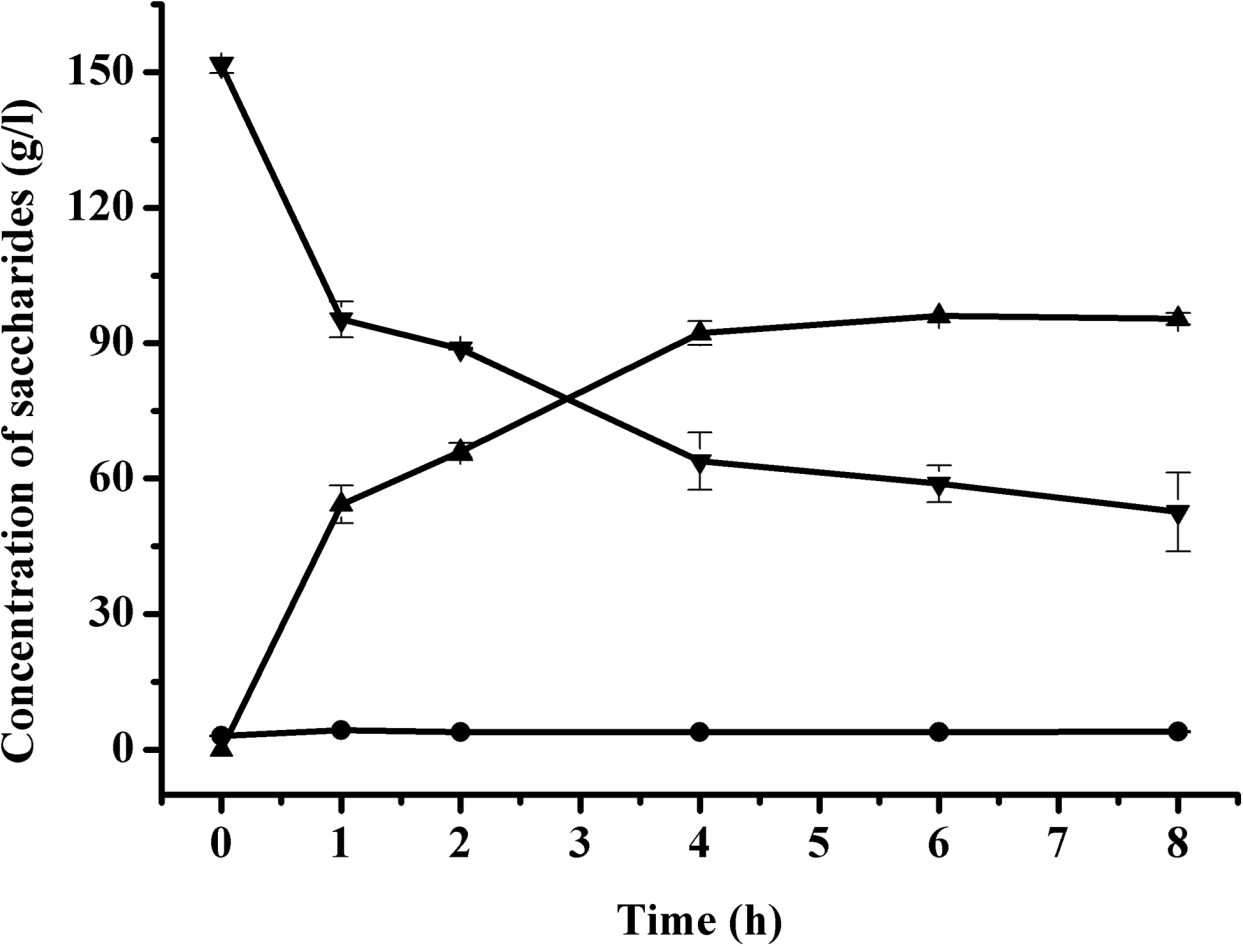
Time course of trehalose production from maltose under optimal conditions. Symbols represent: ▼, maltose; ▲, trehalose; ●, glucose.

Based on the above experiments, the time course of trehalose production from 150 g/l maltose was performed under optimal conditions. As shown in Fig 4, the initial rate of formation of trehalose was faster, and then the rate decreased with time. After 4 h of biotransformation, the concentration of trehalose was 92.2 g/l with a productivity of 23.1 g/(l h) and a yield of 61.5%. And after 6 h of biotransformation, the trehalose concentration reached maximum with a concentration of 96.0 g/l and a productivity of 16.0 g/(l h). Compared with previous reports, the productivity obtained in this study was much higher. At prolonged reaction time, the production intended to reach equilibrium with a small fluctuation because of the reversibility of TreS. In addition, glucose was detected at minimal dose no more than 4 g/l, but it was due to the impurity of lactose instead of lactose hydrolysis, as glucose existed since 0 h (Fig 4).

## Discussion

Up to now, several different substrates, especially low cost substrates, had been employed for trehalose production through various microorganisms and enzymatic routes. With crude glycerol as substrate, 1.56 g/l trehalose was achieved using *Propionibacterium freudenreichii* subsp. *shermanii* under aerobic conditions [28]. Trehalose could also be produced from levulinic acid, a cellulose-derived building block, by *Burkholderia stabilis* [29]. With metabolic engineering *Corynebacterium glutamicum* as biocatalyst, Carpinelli et al. reported biosynthesis of trehalose from maltoheptaose [30]. Similarly to that, Padilla et al. reported trehalose production from glucose by metabolic engineering *C*. *glutamicum* [31]. In addition, instead of microorganisms, three enzymes, amylosucrase, MTSase, and MTHase, were combined for sequential bioconversion of sucrose to trehalose in one-pot [32]. Besides, glycogen and rice hydrolysate could also be used for trehalose production [33, 34]. In consideration of cost and practicability, maltose was more preferred to be selected as substrate in industrial production.

As the concentration and productivity of trehalose produced from maltose by native microbes was rather low, heterologously expression of TreS was a feasible approach to solve this problem. With different purified TreS as biocatalyst, the conversion rate of 59% and 78% was reached at high maltose concentration, respectively, which was much higher than native microbes [15, 35]. However, the purification for TreS was tedious and the thermostability of TreS was unsatisfactory, in the current study, whole cells of engineered *E*. *coli* expressing *treS* was developed as biocatalyst for trehalose production. Furthermore, a dramatic acceleration of maltose conversion was attributed to the increased permeability, which strengthened the advantage of whole-cell biocatalysis as previous reports [36, 37].

For *pseudomonas*, the first reported TreS was identified from *P*. *putida* H262 in 1995. Employing the partial purified TreS, up to 75.2% yield of trehalose was obtained from 50 g/l maltose under pH 7.0 and 20°C after reaction for 24 h [38]. Since then, TreS had been screened from *P*. *putida* H76, *P*. *putida* P06, *P*. *stutzeri* CJ38, etc. All above *pseudomonas* strains were isolated from soil and the corresponding TreS genes were subsequently cloned and expressed. Although those enzymes exhibited different characteristics, all of them gave relatively high activities toward maltose, with a yield of about 40% to 70% under the substrate concentration of 100 g/l to 200 g/l in 24 h [39, 18, 16]. The TreS identified in this study by genome mining also exhibited good activity toward maltose. At pH 8.0 and 45°C, the permeabilized recombinant *E*. *coli* could produce 92.2 g/l trehalose with a yield of 61.5% using 150 g/l maltose as substrate in 4 h. The productivity in this study was much higher than other researches.

Previous studies reported that TreS accomplished both maltose hydrolysis and trehalose formation. As a result, glucose is a common by-product because of the side reaction (Fig 1) [40]. For example, the reaction catalyzed by TreS from *A*. *aurescens* CGMCC 1.1892 gave a glucose yield of 13% from maltose [21]. For the TreS from *T*. *curvata* DSM 43183, a glucose content of 8% was detected after incubation with maltose [17]. Moreover, Tres from *T*. *aquaticus*, *P*. *torridus*, and *E*. *hormaechei* also exhibited hydrolytic activity [19, 20, 22]. The accumulation of glucose would inhibit TreS activity and result in complicated downstream separation [41]. But in this study, no obvious glucose formation was detected during the bioconversion process. Fig 1 showed TreS catalyzed the interconversion of maltose and trehalose. The theoretical equilibrium constant from maltose to trehalose is about 82% using thermodynamic parameters [42]. Therefore, besides further improving the yield of biocatalysis, removing maltose by additional enzymes or certain microorganisms might be beneficial to downstream separation and upscale production.

In conclusion, whole cells of recombinants *E*. *coli* expressing TreS identified from *Pseudomonas* genome exhibited high catalytic ability for trehalose production from maltose. After optimization of the permeabilization and biotransformation conditions, 92.2 g/l trehalose was produced with a high productivity of 23.1 g/(l h) and a yield of 61.5%. No increase of glucose was detected during the process. The biocatalytic process developed in this study might serve as a candidate for the large scale production of trehalose.
